# Supplementary material for: Identification of Genomic Regions Conferring Enhanced Zn and Fe Concentration in Wheat Varieties and Introgression Lines Derived from Wild Relatives
Source: Int J Mol Sci. 2024 Sep 30;25(19):10556. doi: 10.3390/ijms251910556 (PMC11477371; doi:10.3390/ijms251910556)
Supplement: Supplementary file 1 [file ijms-25-10556-s001.zip › ijms-3200858 Supplementary Figures.pdf]

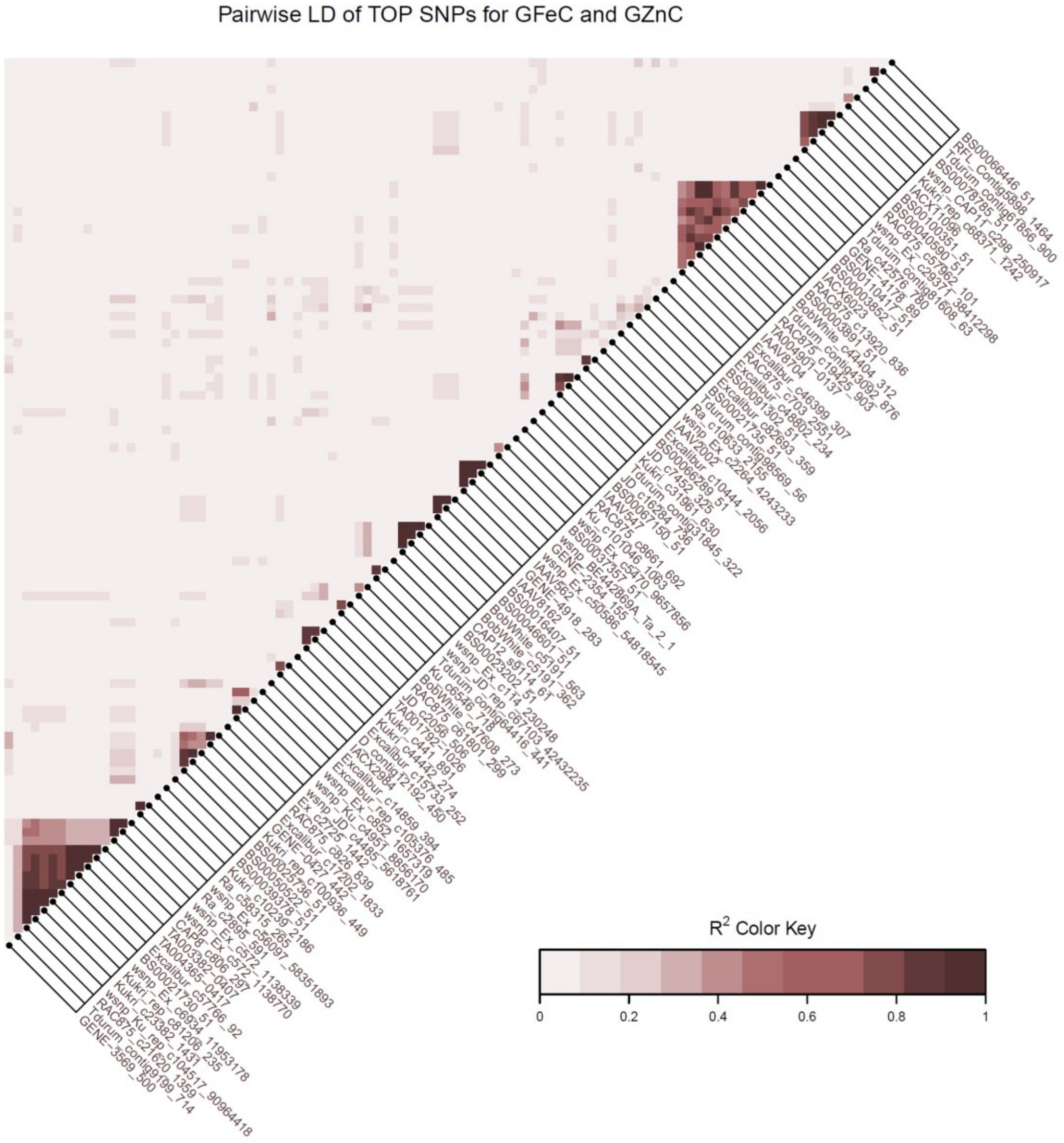

Figure S1. Linkage disequilibrium plot demonstrating correlation (r^2) between each SNP in the association panel consisting of bread wheat varieties, introgression lines and wheat relatives. Markers with high r2 (> 0.8) were considered as an LD block.

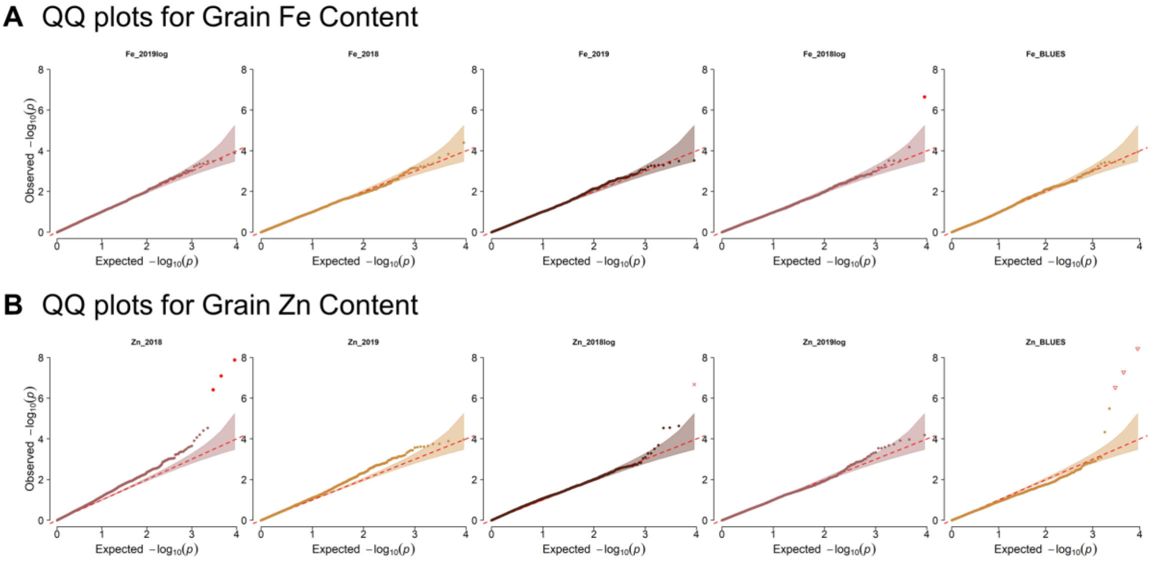

Figure S2. Quantile-Quantile plots of genome-wide association studies results obtained using the BLINK model for Fe (A) and Zn content (B) in common wheat varieties, introgression lines, and wheat relatives.
